# Global health nonsense

**DOI:** 10.1136/bmj.o2932

**Published:** 2022-12-19

**Authors:** Felix Stein, Katerini Tagmatarchi Storeng, Antoine de Bengy Puyvallée

**Affiliations:** 1 Centre for Development and the Environment, University of Oslo; 2 Department of Social Anthropology, University of Cambridge

## Abstract

Global health discourse that either underinforms or misinforms its audience is “global health nonsense.” Such nonsense is widespread, and jeopardises improvement in global health governance, argue **Stein**, **Storeng**, and **de Bengy Puyvallée**

**KEY MESSAGES**

Spin, hyperbole, meaningless buzzwords, and technocratic jargon have become increasingly common in global health discourse. They are part of a broader phenomenon labelled “global health nonsense”
Three main forms of global health nonsense are obfuscation, misrepresentation, and omission of relevant information
Global health nonsense must be called out, because it stifles collective efforts to understand, critically assess, and improve global health governance

One of the most salient features of early 21st century global health discourse is that there is so much nonsense. Spin, hyperbole, meaningless buzzwords, and technocratic jargon have become common fare. Nonsense is not necessarily marked by a will to deceive. Rather, it is characterised by a “lack of a connection to a concern with truth—[an] indifference to how things really are.”^1^

This kind of discourse is marked by its “unclarifiable unclarity”^2^ and tends to be “pointless, unnecessary, or pernicious.”^3^ Whatever the intention behind nonsense may be, it usually underinforms or misinforms its audience, without thereby relying on lies.

Attempts to govern global health according to the goals, actors, modalities, and concepts of financialised markets are partially to blame for the spread of nonsense.^4^ Short term competitive funding rounds, the fetishisation of performance metrics, and a focus on returns on investment increase pressure to constantly project success.^5^ As a result, global health’s leading agencies routinely refer to “accelerators,” “catalysts,” and “investment cases,” emulating the hyperbolic self-praise of Silicon Valley.

Several observers have picked up on this trend and made the case for more meaningful and self-aware discourse. They provide sometimes humorous rejections of vacuous global health speak^6^ ^7^ alongside serious reflection on the way language recapitulates and reinforces existing power hierarchies.^8^ Nevertheless nonsense seems to be proliferating, perhaps because so many of us are implicated in producing it. Taking global public-private partnerships in the response to the covid-19 pandemic as examples, three main forms of global health nonsense are obfuscation, misrepresentation, and the omission of relevant information. We must call out nonsense because it stifles efforts to understand, critically assess, and improve global health governance.

## Obfuscation

Global health nonsense obfuscates reality, often by relying on jargon. Take as an example the Access to Covid-19 Tools Accelerator (ACT-A), which brings together leading global health agencies to speed up the development of and equitable access to covid-19 diagnostics, therapeutics, and vaccines. Jargon obscures the structure of this multibillion dollar health effort, describing ACT-A as an “accelerator,” a “framework,” a “collaboration,” a “partnership,” an “initiative,” or “a platform.” Jargon also obscures how ACT-A works, given that it has a “facilitation council,” “executive hub,” “pillars,” “health systems and response connector,” “pillar leads,” “principals,” “partners,” “key delivery partners,” “co-hosts,” “co-conveners,” “co-chairs,” “sponsors,” and “special envoys.”^9^ Each “pillar,” in turn, has its own “agency leads,” “principals,” “coordinating committees,” “workstreams,” and “workstream leads,” as well as the occasional “shareholders council,” “engagement group,” “investors group,” and “consensus group.”^9^ Many of these terms are “floating signifiers” that obscure more than they elucidate,^10^ papering over the different interests, mandates, degrees of legitimacy, and lines of accountability of ACT-A’s members.^11^ ^12^ Lastly, jargon obscures what will become of ACT-A. While ACT-A promises to continue to “support countries through the transition to long term covid-19 control,” most of its activities are now being “kept warm,” “kept on standby,” “sunset,” or “transitioned” to individual agencies.^13^ The extent to which these are euphemisms for a simple end to ACT-A is unclear.

## Misrepresentation

Global health nonsense also misrepresents reality through words, diagrams, or metrics.^14^ A good example is the number of vaccine doses delivered by ACT-A’s “vaccine pillar” Covax. Covax initially promised to provide “access to at least two billion doses of safe and effective covid-19 vaccines to the most vulnerable [by the end of 2021]”^15^ but ended up delivering less than half that (832.5 million).^16^ While it was accused of failing at its mission, Covax celebrated “700 million doses delivered,” and “one billion doses delivered” in early 2022 as “historic” successes, complete with videos of people fist pumping in joy over the arrival of vaccine shipments. Further, Covax and the countries that donated vaccines to it opted to highlight whatever metric best portrayed their impact. They sometimes emphasised “pledged doses” and sometimes “secured doses,” occasionally “ordered doses” and “delivered doses,” but all too rarely “administrated doses.” This led *Politico* to conclude that “a dose is not a dose” in the context of Covax’s vaccine rollout.^17^

Another metric that subtly misrepresents reality in favour of global public-private partnerships like Gavi, the Vaccine Alliance and the Global Fund to Fight AIDS, Tuberculosis, and Malaria is the number of “lives saved.” This emotionally appealing metric is characterised by high uncertainty, is prone to overestimation, and tends to misattribute positive health outcomes to individual programmes, rather than the host of institutions and interventions involved in bringing them about.^18^ It can even lead to double reporting as a person co-infected with AIDS and tuberculosis who receives treatment for both can be counted as though “two lives” had been saved. As with Covax’s vaccine doses, “lives saved” blurs marketing with unbiased reporting of global health results.^19^ It embellishes the truth to reinforce vertical programming, distorting national health priorities and budgets in the process.^18^

## Omitting relevant information

A final form of global health nonsense is to leave out relevant information, such as frank discussions of political and economic choices, challenges, and shortcomings. Leaders of high income countries and public-private partnerships repeatedly insisted on the importance of multilateralism, the urgency of global vaccine equity, and the truism that “nobody is safe until everyone is safe.” They often made such generic points instead of discussing concrete matters like vaccine hoarding; soaring prices for covid-19 diagnostics, treatments, and vaccines; the limits of intellectual property in pandemic times; how publicly funded public–private partnerships spend their budgets; or what exactly the public should expect in return for subsidising the pharmaceutical industry in times of crisis.^11^

Similarly, in the autumn of 2022, the head of the World Bank argued that its new financial intermediary fund for pandemic prevention, preparedness, and response (FIF) would “complement” existing global health institutions, “catalyse investments,” and “serve as an integrator” rather than a new silo.^20^ But he did not tackle concrete concerns that FIF competes with existing global health funds and institutions, that it should broaden its base of participating countries,^21^ or that its claim to provide “catalytic” funding remains to be substantiated.

## Conclusion

The examples of nonsense we have identified will be recognisable to many in the global health community. A certain amount of obfuscation, misrepresentation, and omission may be unavoidable, but it is not innocuous. By fostering “strategic ignorance,”^22^ nonsense stifles collective efforts to understand, assess, debate, and improve global health governance. Indeed, our acceptance of nonsense made it possible for global health leaders to at once claim that we “accelerated vaccine equity” while also maintaining “vaccine apartheid.” Crucially, nonsense contributes to the inequity laid bare in the global response to the covid-19 pandemic.

As global health research, publishing, and policy become more reliant on a smaller number of funders, it gets increasingly difficult to conduct and publish independent analyses of policy initiatives.^23^ Challenging the status quo can mean facing ridicule, censorship, or exclusion from the centres of epistemic and economic power. For example, in a 2021 interview, Bill Gates, whose foundation funds all major ACT-A agencies, responded to the proposal of a temporary waiver of intellectual property rights to increase access to covid-19 vaccines by calling it “the stupidest thing [he] ever heard.”^24^

We are all implicated in the nonsense that permeates global health: policy makers, think tanks, consultants, non-governmental organisations, and universities are increasingly compelled to project success to attract funding and garner influence. Stuck in a “success cartel,”^19^ we risk reinforcing the power asymmetries that undermine health equity.^23^ ^25^ All of us therefore need to find the courage to avoid, identify, and call out hogwash when we hear it. It’s time to cut the global health nonsense.

**Figure:**
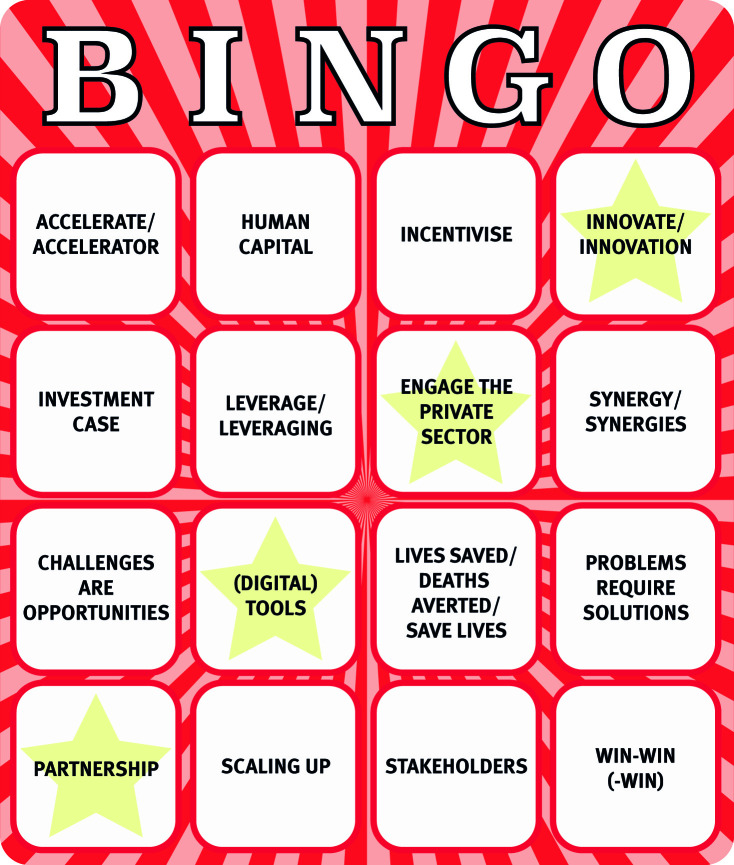
Buzzword Bingo: To support readers in calling out nonsense, we suggest they play Buzzword Bingo in their next global health meeting. Put a cross on the square when you hear the terms in question. Whoever fills a horizontal, vertical, or full diagonal row first wins!

## Notes

FS is an economic anthropologist, who specialises in global health financing. He provided the basic structure of the article and drafted much of it. KTS is a medical anthropologist whose research focuses on the politics of global health. She brought the initiative for writing the article and co-drafted it. ABP is a politics and governance expert with a background in international relations. He helped draft the article and provided valuable empirical and conceptual input. All three authors collected data and literature.

## Notes

Provenance: not commissioned, externally peer reviewed.
